# Herbivory by leaf-cutter ants changes the glandular trichomes density and the volatile components in an aromatic plant model

**DOI:** 10.1093/aobpla/plx057

**Published:** 2017-10-24

**Authors:** Luiz Ricardo dos Santos Tozin, Marcia Ortiz Mayo Marques, Tatiane Maria Rodrigues

**Affiliations:** Department of Botany, São Paulo State University (UNESP), Institute of Biosciences of Botucatu (IBB), Botucatu, SP 18618-970, Brazil; Instituto Agronômico (IAC), Laboratory of Natural Products, Campinas, SP 13020-902, Brazil

**Keywords:** *Acromyrmex rugosus*, gas chromatography, herbivore-induced plant volatiles (HIPVs), *Ocimum*, terpenes

## Abstract

Herbivory can induce several structural and functional alterations in the plant secretory system. Glandular trichomes are the main sites of production of volatile organic compounds (VOCs) with several chemical properties in Lamiaceae species. *Ocimum* species usually have three morphotypes of glandular trichomes (morphotype I is peltate and has a wide four-celled head; morphotype II is capitate and has a unicellular head; and morphotype III is capitate with a bicellular head) which produce a great amount of terpenes, although other chemical categories of substances are also produced. Despite the abundance of trichomes producing important anti-herbivory components in their leaves, the association between *Ocimum* species and leaf-cutter ants has been commonly registered in Brazil. We investigated the effect of leaf-cutter ant attack on the density of the glandular trichomes and on the chemistry of the VOCs released from leaves of *O. gratissimum*. Plants were subjected to *Acromyrmex rugosus* attack until 90 % of leaves were removed. After 40 days from the leaf-cutter attack, both treatments were sampled. The glandular trichome density was analysed by scanning electron microscopy. The VOCs were extracted utilizing headspace solid-phase microextraction (HS-SPME) technique and analysed by gas chromatography. Generally, the density of glandular trichomes increased in the adaxial leaf surface of the attacked plants. However, we bring novelties on this topic since we analysed the density of each morphotype separately. The morphotype I decreased in the abaxial leaf surface, and increased in the adaxial leaf surface; the morphotype II increased in both leaf surfaces; and the morphotype III decreased in the abaxial leaf surface and remained constant in the adaxial leaf surface of attacked plants. In leaves of attacked plants, the (*Z*)-β-ocimene increased by 50 %, the α-selinene by 13 % and the germacrene D by 126 %, whereas the eugenol decreased by 70 %. Our data point to a differential response of each glandular morphotype in *O. gratissimum* and are consistent with the idea of a compartmentalization of functions among the different glandular morphotypes in the plant defence against environmental factors.

## Teaser

Leaf-cutter ants are the most important pest insect in agriculture. Their association with Ocimum species is common in Brazil. O. gratissimum presents three morphotypes of glandular trichomes producing compounds with anti-herbivore properties. Under an experimental approach, we found significant alterations in O. gratissimum volatile oil composition after leaf-cutter ant attacks. The total density of glandular trichomes increased in attacked plants; however, we bring novelties in the sense that we observed differential responses to each glandular morphotype. This could be related to the specific substances produced by each trichome morphotype, contributing to the idea of functional compartmentalization in plant defense.

## Introduction

Glandular trichomes can be viewed as a combination of structural and chemical defences against several abiotic and biotic factors (Werker 2000; Tozin *et al.* 2015a), such as herbivory. These defences are due the fact that glandular trichomes form a protect layer around the leaves (Werker 2000), and produce substances that can be poisonous or repellent to herbivorous organisms (Dalin *et al.* 2008). Several plant species respond to damage caused by herbivores by producing new leaves with an increased density of trichomes. The magnitude of the reported increase in trichome density is commonly between 25 and 100 %, but in some cases it is as large as 500–1000 % (Dalin *et al.* 2008). Such responses involving changes in density of trichomes are expressed within days or weeks (Baur *et al.* 1991; Agrawal 2000; Rautio *et al.* 2002).

A wide range of plant species have been shown to emit new herbivore-induced plant volatiles (HIPVs) or increased amounts of constitutive HIPVs from glandular trichomes following herbivore damage (Dicke *et al.* 2003; Unsicker *et al.* 2009). Some species exhibit a rapid response to herbivory attacks, and the HIPVs can be detected in the first 45 min after feeding (Kigathi *et al.* 2009).

The effect of a herbivore attack on trichome production may vary according to the identity of the herbivore (Dalin *et al.* 2008), and not all herbivorous insects induce changes in trichome production (Traw and Dawson 2002). Different species of herbivores could induce different responses due to differences in substances present in their saliva (Turlings *et al.* 1993; Geervliet *et al.* 1997). Herbivory caused by leaf-cutter ants is known to cause losses in agriculture estimated in the billions of dollars (Hölldobler and Wilson 1990). The leaf-cutter ants are the most important pest insect in agriculture (Zanuncio *et al.* 1996) and cultivated forest of the Neotropic (Della Lucia *et al.* 1993). *Acromyrmex* ants are among the most damaging invertebrates in the Neotropic (Boulogne *et al.* 2012) and have been registered in different regions of America (Farji-Brener and Ruggiero 1994). In Brazil, *Acromyrmex* ants have been commonly found in several States and their association with different plants with economic importance, including *Ocimum* species, is well known (Rando and Forti 2005).

*Ocimum* genus comprises aromatic species of Lamiaceae with particular importance in the medicine and culinary due to the production of essential oils (Silva *et al.* 2008). Such species are featured by the presence of three morphotypes of glandular trichomes with differential morphological and functional aspects (Werker *et al.* 1993; Naidoo *et al.* 2013). The morphotype I is peltate and has a wide four-celled head; the morphotype II is capitate and has a unicellular head; and the morphotype III is capitate and has a bicellular head (Werker *et al.* 1993; Naidoo *et al.* 2013). In addition to their morphological and subcellular peculiarities, differences in the composition of the produced secretion have been histochemically detected in each one of these glandular morphotypes (Naidoo *et al.* 2013).

*Ocimum gratissimum* presents these three morphotypes of glandular trichomes in their leaves, and is important for the production of essential oils widely exploited by the pharmaceutical industry, like eugenol (Silva *et al.* 1999; Fernandes *et al.* 2013) and 1,8-cineole (Silva *et al.* 1999), among others. In addition, *O. gratissimum* is cultivated in gardens, and is used in the popular medicine to treat rheumatism, paralysis, epilepsy and mental illness, and contains biologically active substances with insecticide, nematicide, fungicide and antimicrobial proven properties (Efraim 2001).

In this paper, we investigated the effect of leaf-cutter ant attacks on the density of the glandular trichomes and on the VOCs chemical composition in leaves of *O. gratissimum*. We hypothesized that the plant attack by leaf-cut ants could induce the increase in the glandular trichome density and lead to the production of different volatile compounds in an effort of an incremental protection against future attacks.

## Methods

### Plant material

For cuttings, stem fragments of *O. gratissimum* ~10 cm long were collected from six individuals growing in the medicinal garden belonging to Sao Paulo State University (UNESP), in Botucatu municipality (22°52 S, 48°26 W), in central-west region of São Paulo State, in south-eastern Brazil. The stem cuts were placed in trays containing commercial substrate (Bioplant®, Nova Ponte, Minas Gerais, Brazil) and 20 individuals were obtained. The plants were maintained under humid conditions until rooting. After 40 days, the plant saplings were transferred to 6.0 L pots filled with complete Hoagland and Arnon’s (1950) n^o^. 2 nutrient solution with 75 % ionic concentration. The pots were kept in a greenhouse under mean maximum and minimum air temperatures of 26.5 and 21.5 °C, respectively, and mean relative humidity of 70 %. The solutions were prepared using deionized water and were constantly aerated and renewed every week to minimize nutrient depletion or pH changes.

Vouchers were deposited in the Irina Delanova Gemtchújnicov Herbarium (BOTU), of the Department of Botany, Institute of Biosciences of Botucatu, IBB, UNESP, Botucatu, Sao Paulo, Brazil, under registration number 32798.

### Experimental design

**Figure 1.**
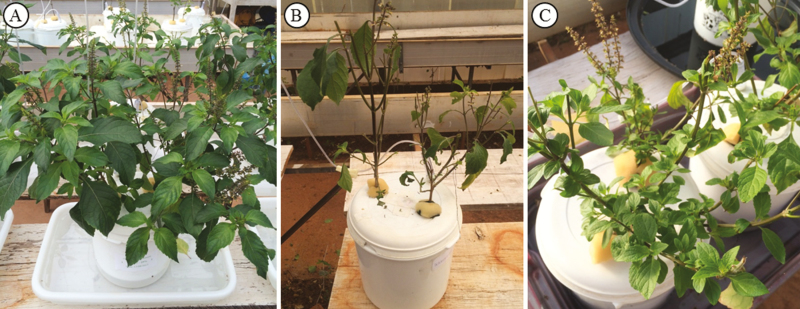
Experimental design of *Ocimum gratissimum* submitted to *Acromyrmex rugosus* attack. (A) Control plants. (B) Attacked plants by *A. rugosus*. (C) Attacked plants after 40 days.

At 65 days after transplanting to the hydroponic system, the plants were placed in a greenhouse naturally infested by leaf-cutter ants. The experiment consisted of two treatments, a control (protected from ant attacks; *n* = 8) (Fig. 1A) and attacked plants (*n* = 8). The pots with control plants were placed inside a container filled with water and dishwashing detergent (that surrounded the pots without enter them), to avoid the ant approach. The attacked plants were exposed to *Acromyrmex rugosus* (leaf-cutter ant) for 48 h, until 90 % of leaves were removed by the herbivores (Fig. 1B). Posteriorly, the pots were arranged in a randomized design in a greenhouse, and the apical meristems were labelled to ensure that the analysed leaves were produced at the same time. After 40 days (Fig. 1C), the fully expanded leaves that were formed after the herbivore attacks and leaves from control plants were collected to analyse the density of glandular trichomes and the volatile components chemical composition. To analyse the glandular trichomes density, we collected leaves with the same size produced at the same time.

### Density of glandular trichomes

To evaluate the glandular trichomes density, eight leaves of each treatment (two leaves per plant, being one for each leaf surface analysis) were fixed in 2.5 % glutaraldehyde with 0.1 M phosphate buffer, pH 7.3, overnight at 4 °C, dehydrated in a graduated acetone series, critical-point dried, mounted on aluminium stubs, and gold-coated (Robards 1978). The material was examined with a FEI Quanta™ scanning electron microscope.

The glandular density in leaves was calculated in 1 mm^2^ using the Scandium software with an image-capture system coupled to the scanning electron microscope. The density of all glandular trichomes and of each morphotype was calculated in the middle region of the leaf blade, in both abaxial and adaxial leaf surface.

### Extraction and analysis of volatile organic compounds

The extraction and analysis of volatile organic compounds (VOCs) were conducted in leaf samples collected from both treatments (*n* = 8).

The headspace solid-phase microextraction (HS-SPME) technique was used to extract the volatile compound. For capture of the volatile constituents, 0.45 g of leaves were placed in clear screw cap vials with 15 mL of deionized water. The vial was placed in water bath at 95 °C for 1 h. The volatile phase was exposed to CAR/PDMS fibre (Carboxen/Polydimethylsiloxane, film thickness 75 μm, Supelco, Bellefonte, PA, USA) for 15 min.

The qualitative and quantitative (area normalization method) analyses of the volatile components were performed on a gas chromatograph coupled to a mass spectrometer (CG-MS, Shimadzu, QP-5000), with an OV-5 fused silica capillary column (30 m × 0.25 mm × 0.25 μm, Ohio Valley Specialty Chemical, Inc.), operating at an MS ionization voltage of 70 eV, with helium as the carrier gas (1.0 mL min^−1^). The following chromatography conditions were used: injector at 240 °C, detector at 230 °C, split 1/20, and the temperature programme: 50 °C, 2 min; 50–180 °C, 3 °C min^−1^; 180–230 °C, 10 °C min^−1^. The compounds were identified by comparison of the acquired mass spectra with those stored in the GC/MS database of the system (NIST 62 lib.) and retention indices (RI; Adams 2007). The RI were obtained from the injection of a mixture of *n*-alcanes (Sigma-Aldrich, C9-C24), employing the same temperature programme conditions described above for GC/MS, applying the equation of Van den Dool and Kratz (1963). For each sample the fibre was conditioned in GC-FID (Shimadzu, GC-2010/AOC-20i) with the temperature programme: 50–240 °C, 7 °C min^−1^.

### Statistical analyses

Data referring to the total density of glandular trichomes, the relative density of each glandular morphotype and the concentration of each volatile compound in the essential oil were tested by one-way ANOVA. Tukey test (*P* ˂ 0.05) was conducted to compare the different treatments.

## Results

### Density of glandular trichomes

**Figure 2.**
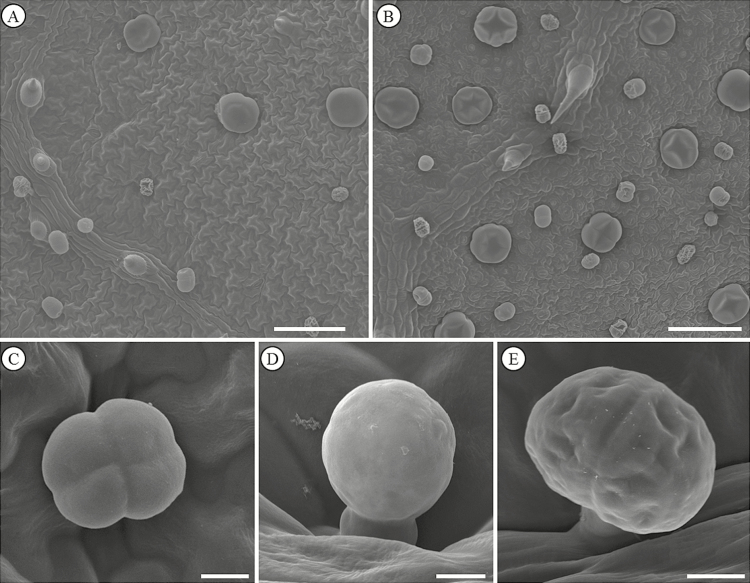
Scanning electron micrographs of adaxial (A) and abaxial (B) leaf surface of *Ocimum gratissimum*. (C–E) Glandular trichomes of *O. gratissimum.* (C) Morphotype I. (D) Morphotype II. (E) Morphotype III. Scale bars A–B = 100 µm; C–E = 10 µm.

**Figure 3.**
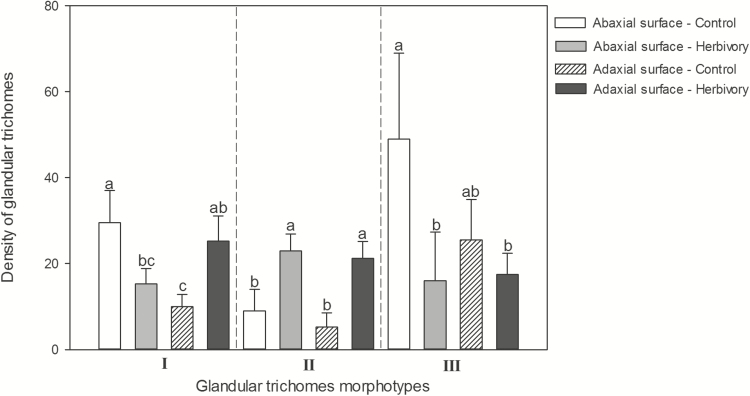
Density (mm^2^) of each morphotype of glandular trichomes on both leaf surfaces of *Ocimum gratissimun* submitted to *Acromyrmex rugosus* attack and control. Means followed by different letters indicate statistical differences in the glandular density among treatments and leaf surfaces in each morphotype (Tukey test *P* < 0.05).

Both leaf surfaces of *O. gratissimum* (Fig. 2A and B) presented three morphotypes of glandular trichomes (Fig. 2C–E) occurring side by side. The glandular trichome density increased in the adaxial leaf surface in the attacked plants (*P* = 0.035; *F*_1, 14_ = 7.332) and did not vary in the abaxial leaf surface (*P* = 0.085; *F*_1, 14_ = 4.253) in plants attacked by ants. However, we registered a differential response of each glandular morphotype to the ant attacks. In the attacked plants, the density of the morphotype I decreased in the abaxial leaf surface and increased in the adaxial leaf surface of attacked plants (*P* < 0.001; *F*_3, 28_ = 11.429); the density of the morphotyte II increased in both leaf surfaces (*P* < 0.001; *F*_3, 28_ = 18.732); and, in the morphotype III, the density decreased in the abaxial leaf surface and remained constant in the adaxial leaf surface (*P* = 0.011; *F*_3, 28_ = 6.152) (Fig. 3).

### Volatile organic compounds

Twenty-nine compounds were chemically identified in control plants of *O. gratissimum* and 20 in individuals submitted to ant attacks. In both cases the compounds identified represented ~97 % of volatile components present in the chromatogram. Overall, the major compounds identified were 1,8-cineole (25.79 %), β-selinene (17.39 %), eugenol (16.61 %), (*E*)-caryophyllene (11.13 %), (*Z*)-β-ocimene (6.38 %) and α-selinene (4.55 %) (Table 1). In the attacked plants the (*Z*)-β-ocimene increased by 50 %, the α-selinene by 13 % and the germacrene D by 126 %, whereas the eugenol decreased by 70 % (Table 1). In addition, the attack by *A. rugosus* promoted the increase of five minority substances and the decrease of three minority substances in the leaves of *O. gratissimum* (Table 1).

**Table 1.** Chemical composition of volatile organic components (%) from leaves of *Ocimum gratissimum* submitted to *Acromyrmex rugosus* attack and control. RI = retention index calculated; RI* = retention index (Adams 2007); *indicates substances with statistical difference; means followed by different letters indicate statistical differences (Tukey test *P* < 0.05).

Component	Control	Herbivory	*P*	*F*_1, 6_	RI	RI*
Hydrocarbon monoterpenes						
(*Z*)-β-Ocimene*	6.38 b	12.07 a	0.043	8.617	1041	1037
β-Pinene	2.22 a	2.41 a	0.494	0.566	976	979
Myrcene*	1.11 b	3.54 a	0.007	26.151	993	990
Sabinene	0.62 a	0.88 a	0.130	3.616	974	975
α-Pinene	0.52 a	0.61 a	0.356	1.086	933	939
γ-Terpinene*	0.29 a	0.00 b	0.007	26.01	1060	1059
(*E*)-β-Ocimene*	0.21 b	0.69 a	0.006	28.981	1051	1050
ρ-Cymene	0.14 a	0.00 a	0.117	3.973	1025	1024
α-Terpinene	0.06 a	0.00 a	0.374	1.000	1009	1017
Oxygenated monoterpenes						
1,8-Cineole	25.79 a	21.38 a	0.074	5.802	1034	1031
Eugenol*	16.61 a	5.1 b	0.001	60.465	1361	1359
α-Terpinol*	0.36 a	0.00 b	<0.001	165.753	1192	1188
Hydrogenated sesquiterpenes						
β-Selinene	17.39 a	18.79 a	0.276	1.592	1493	1485
(*E*)-Caryophyllene	11.13 a	12.91	0.432	0.762	1425	1419
α-Selinene*	4.55 b	5.73 a	0.014	17.753	1501	1498
Germacrene D*	1.86 b	4.21 a	0.044	8.467	1486	1485
α-Humulene	1.54 a	2.12 a	0.058	6.917	1458	1454
α-Copaene	1.29 a	1.70 a	0.152	5.125	1381	1376
7-*epi*-α-Selinene*	1.05 b	1.44 a	0.005	31.469	1522	1522
α-*trans*-Bergamotene	0.78 a	1.22 a	0.268	1.654	1441	1434
allo-Aromadendrene*	0.76 a	0.00 b	<0.001	144.069	1465	1460
β-Elemene*	0.41 b	1.26	0.034	28.136	1396	1390
δ-Cadinene*	0.38 b	0.57 a	0.015	16.770	1528	1523
α-Neocallitropsene	0.12 a	0.00 a	0.119	3.923	1482	1476
β-Cubebene	0.09 a	0.00 a	0.374	1.000	1395	1388
α-Guaiene	0.05 a	0.00 a	0.374	1.000	1443	1439
β-Copaene	0.03 a	0.00 a	0.374	1.000	1434	1432
Oxigenated sesquiterpenes						
Caryophyllene oxide	0.81 a	0.26 a	0.119	3.900	1586	1583
β-Bourboneno	0.03 a	0.16 a	0.482	0.601	1389	1388
Hydrocarbon monoterpenes*	3.85 b	20.20 a	0.026	14.046		
Oxygenated monoterpenes*	42.76 a	21.38 b	0.002	47.407		
Hydrogenated sesquiterpenes	33.17 a	49.94 a	0.073	5.820		
Oxigenated sesquiterpenes	0.84 a	0.42 a	0.155	3.056		
Total identified	96.57	97.04				

In a general way, the hydrocarbon monoterpenes increased, and the oxygenated monoterpenes decreased in the VOCs of leaves from attacked *O. gratissimum* plants (Table 1).

## Discussion

Our data showed a differential response of each glandular morphotype in leaves of *O. gratissimum* plants attacked by *A. rugosus*; in addition, significant alterations were registered in the composition of the VOCs of attacked plants. Our work is novel in the sense that we presented the density alterations to each glandular morphotype separately. All studies present in the literature report the total density of glandular trichomes (Molina-Montenegro *et al.* 2006; Dalin *et al.* 2008), and this could not show what happens with each glandular trichomes.

The increasing of density of glandular trichome after attack of herbivores has been demonstrated in different groups of plants (Levin 1973; Dalin *et al.* 2008), but not in each glandular morphotype individually. The differential variation of the glandular morphotypes density could be related to the specific substances produced by each morphotype. Several studies have demonstrated that different morphotypes of glandular trichomes could produce secretion with a different chemical nature (Fahn 1979; Tozin *et al.* 2015a; Silva *et al.* 2016). In *Ocimum* species, studies have also demonstrated histochemical variations in the substances produced by each morphotype of glandular trichomes (Naidoo *et al.* 2013). Naidoo *et al.* (2013) have showed that for *O. obovatum*, peltate trichomes (morphotype I) are more active in the production of lipophilic substances than capitate trichomes (morphotypes II and III). At the same time, whether the essential oil composition produced by each morphotype of glandular trichome varies need to be better investigated. New research is being conducted to identify the chemical composition of the volatile components produced by each morphotype of glandular trichomes. This research involves using of techniques of microdissection and microextration to search for an association between the density of each morphotype of glandular trichome and volatile components composition produced. In a general way, the increase of HIPV, such as germacrene D and (*Z*)-β-ocimene, was significant in plants attacked by *A. rugosus* and can be related to the attraction of generalist natural enemies of herbivores (Turlings *et al.* 1998; Dudareva *et al.* 2003; De Boer and Dicke 2004), or can be associated with the production of substances which act as insecticide or astringent (Kiran and Devi 2007; Tozin *et al.* 2015b). The increasing in β-ocimene production has been demonstrated after spider mite attacks in *Lotus japonicus* (Arimura *et al.* 2004). The germacrene D, which increased by 126 %, is known by its insecticidal activity against mosquitoes (Kiran and Devi 2007) and repellent role against ticks (Birkett *et al.* 2008) and aphids (Bruce *et al.* 2005). In addition, germacrene D plays a role as a precursor of several sesquiterpenes, such as selinenes (Bülow *et al.* 2000; Telascrea *et al.* 2007). So, the increasing of α-selinene can be associated with the increasing of germacrene D; and the role of α-selinene in plant defence must be better understood.

Eugenol is known as a toxicant, antifeedant, deterrant, irritant and repellant chemical substance (Tan and Nishida 2012). Nevertheless, some insect species are known to be attracted to eugenol for unknown reasons (Tan and Nishida 2012). Considering that the eugenol decreased by 70 % in *O. gratissimum* after leaf-cutter ant attack by *A. rugosus*, we could suggest that *A. rugosus* seems to be an example of these insects. However, new experimental tests are required to confirm this hypothesis.

Whether the compounds produced by the new leaves would prevent new leaf-cutter ants attack in *O. gratissimum* remains unknown. However, Kost *et al.* (2011) tested the ‘induced defence hypothesis’ in lima bean and provided the first empirical evidence that the foraging behaviour of leaf-cutter ants is affected by the VOCs after herbivory. In addition, Thiele *et al.* (2014) showed that the leaf-cutter ants could exhibit a delay to reject leaves with new VOCs profile. The leaf-cutter ants may spend 5 days until they detect the new VOCs produced. This happens because the new VOCs could present antifungal properties and avoid the fungal grow in the ants colonies (Thiele *et al.* 2014). New works should be conduct to investigate the ants’ foraging behaviour in the leaves developed after herbivory in *O. gratissimum*.

## Conclusions

Our work showed that the three morphotypes of glandular trichomes of *O. gratissimum* respond differently to leaf-cutter ant attacks, and the volatile organic components are changed after herbivory. Our findings lead to the idea of a compartmentalization of functions among the different glandular morphotypes in the plant defence against environmental factors. If this hypothesis is confirmed, this work can support future studies concerning plant breeding, to produce plants more resistant.

## Sources of Funding

L.R.S.T. received a doctoral scholarship from CAPES; and M.O.M.M. received a productivity scholarship in research from CNPq.

## Contributions by the Authors

L.R.S.T. and T.M.R. conceived the idea; L.R.S.T. designed and conducted the experiments; all authors analysed and discussed the results; L.R.S.T. and T.M.R. wrote the manuscript; and M.O.M.M. commented on the manuscript.

## Conflicts of Interest

None declared.
